# Microbial Competition of *Rhodotorula mucilaginosa UANL-001L* and *E*. *coli* increase biosynthesis of Non-Toxic Exopolysaccharide with Applications as a Wide-Spectrum Antimicrobial

**DOI:** 10.1038/s41598-017-17908-8

**Published:** 2018-01-15

**Authors:** Augusto Vazquez-Rodriguez, Ximena G Vasto-Anzaldo, Daniel Barboza Perez, Eduardo Vázquez-Garza, Héctor Chapoy-Villanueva, Gerardo García-Rivas, Javier A. Garza-Cervantes, Jéssica J. Gómez-Lugo, Alma Elizabeth Gomez-Loredo, Maria Teresa Garza Gonzalez, Xristo Zarate, Jose Ruben Morones-Ramirez

**Affiliations:** 1 Universidad Autónoma de Nuevo León, Facultad de Ciencias Químicas, Pedro de Alba, S/N, San Nicolas de los Garza, Nuevo León Mexico; 2 Centro de Investigacion en Biotecnologia y Nanotoxicologia, Facultad de Ciencias Quimicas, Universidad Autonoma de Nuevo Leon. Parque de Investigacion e Innovacion Tecnologica, Km. 10 autopista al Aeropuerto Internacional Mariano Escobedo, Apodaca, Nuevo Leon 66629 Mexico; 3 Universidad Autónoma de Nuevo León, Facultad de Ciencias Biológicas, Pedro de Alba, S/N, San Nicolas de los Garza, Nuevo León Mexico; 4 Cátedra de Cardiología y Medicina Vascular. Escuela de Medicina. Tecnologico de Monterrey, Monterrey, Nuevo León Mexico; 5 Centro de Investigación Biomédica. Hospital Zambrano-Hellion. Tecnologico de Monterrey, San Pedro Garza-Garcia, Nuevo León Mexico

## Abstract

Bacterial species are able to colonize and establish communities in biotic and abiotic surfaces. Moreover, within the past five decades, incidence of bacterial strains resistant to currently used antibiotics has increased dramatically. This has led to diverse health issues and economical losses for different industries. Therefore, there is a latent need to develop new and more efficient antimicrobials. This work reports an increased production of an exopolysaccharide in a native yeast strain isolated from the Mexican Northeast, *Rhodotorula mucilaginosa* UANL-001L, when co-cultured with *E*. *coli*. The exopolysaccharide produced is chemically and physically characterized and its applications as an antimicrobial and antibiofilm are explored. The exopolysaccharide is capable of inhibiting planktonic growth and biofilm formation in *Escherichia coli*, *Pseudomonas aeruginosa and Staphylococcus aureus*. Additionally, the exopolysaccharide studied here does not exhibit cytotoxic effects when assessed both, *in vitro* against an H9c2 mammalian cell line, and *in vivo* in a murine toxicity model. Taken together, the properties of this exopolysaccharide indicate that it has potential applications to inhibit bacterial colonization in medical and industrial settlings.

## Introduction

Within the last five decades there has been an increased frequency in the emergence of bacterial strains resistant to commercially available antibiotics^1–4^. Therefore, there is an urgent need to seek, develop and design new antimicrobials to treat infections and to combat bacterial strains in industrial settings^5^. Bacteria have the ability to colonize biotic and abiotic environments through the formation of biofilms^6^. It has been estimated that 80% of bacterial infections in humans are caused by bacterial biofilms, and 50% of the nosocomial infections are acquired due to biofilm-infected medical devices^7^. Moreover, the presence of biofilms is one of the leading causes of recurring infections and emergence of resistant strains, which has lead to both health issues and large economical losses^8^. It is therefore imperative to develop new antimicrobials with the ability to control both planktonically grown bacteria and those found within biofilms^9,10^.

In nature, microorganisms establish complex communities that communicate and interact through diverse beneficial and antagonistic interactions. Antagonistic interactions in many cases involve the production of a myriad of compounds that induce microbial inhibition and bacterial cell damage^11^. Fungi are of particular interest since these microorganisms are usually capable of producing a broad range of metabolites that allow them to modify the physicochemical properties of their surroundings and control populations of their bacterial neighbors^12^. Particularly, many yeasts are able to synthetize and secrete exopolysaccharides (EPS), biopolymers comprised of different monosugar units that present different conformations and structures^13–15^. It has been observed that the biological function of EPS is closely related to their configuration and one of the biological functions found in some exopolysaccharides is their ability to inhibit bacterial growth^16–19^.

EPS are mainly produced when microbial cells are under stressful conditions, since their overproduction is a defense mechanism against different microorganisms and toxins^20^. Particularly, EPS act as a matrix for biofilm formation, offering a protective barrier against physical and chemical stressors. Additionally, in rare occasions, EPS can act as molecules that inhibit the biofilm formation process of competing microorganisms^21,22^.

Recently, diverse authors have identified and isolated a variety of exopolysaccharides from different microorganisms, such as microalgae, bacteria, plants and fungi. Some of these EPS exhibit antimicrobial and antibiofilm activity when tested against distinct bacterial strains^23,24^. It is therefore of interest to study the properties of novel EPS produced by different microbial species, since, in some cases, they are capable of controlling microbial proliferation and biofilm formation.

Our research group recently isolated a metal-resistant *Rhodotorula mucilaginosa* UANL-001L yeast strain from an industrial water effluent contaminated with different heavy metals^25^. The strain was found to be capable of producing higher EPS yields under metal-stress conditions^25^. In this work, we find that *Rhodotorula mucilaginosa* UANL-001L increases EPS biosynthesis, when co-cultured with *E*. *coli*. We therefore suggested that this increased production could be linked to a defense mechanism in *Rhodotorula mucilaginosa* UANL-001L. This led us to explore antimicrobial and antibiofilm properties of the produced EPS. We report here that the EPS produced by the autochthonous strain exhibit interesting antimicrobial and antibiofilm properties against different Gram-negative and Gram-positive organisms. The EPS produced are chemically and physically characterized and, to dimension their possible applications in industry and as therapeutic agents, their cytotoxic effects are assessed, *in vitro* using mammalian cell cultures and *in vivo* through a murine toxicity model.

## Material and Methods

### Growth Conditions of Different Strains Used

The yeast strain used in this work was *Rhodotorula mucilaginosa* UANL-001L. The strain was isolated from the water streams of the Pesqueria River, located in the state of Nuevo Leon in the Northeast of Mexico. The inoculum was conserved at −80 °C in YM broth (Difco, BD) supplemented with 20% (V/V) glycerol.

Three exploratory media cultures were tested: Yeast Mold (YM), Yeast Mold Mineral (YMM) and YMMZ (Yeast Mold Mineral with added Zn). The specific compositions of each of the media are displayed in Table 1.

**Table 1:** Composition of the different tested to grow *R*. *mucilaginosa* UANL-001L.

Yeast Mold Media (YM)		Yeast Mold Mineral Media (YMM)		Yeast Mold Mineral Media with added Zn (YMMZ)	
*Ingredient*	*Conc*. *(g/L)*	*Ingredient*	*Conc*. *(g/L)*	*Ingredient*	*Conc*.*(g/L)*
*Yeast Extract*	5	*Yeast Extract*	2	*Yeast Extract*	2
*Dextrose*	20	*Dextrose*	20	*Dextrose*	20
*Malt Extract*	3	*KH*_2_ *PO*_4_	1	*KH*_2_ *PO*_4_	1
*Peptone*	5	*NaCl*	0.1	*NaCl*	0.1
		*MgSO*_4_ *7H*_2_ *O*	0.5	*MgSO*_4_ *7H*_2_ *O*	0.5
		*CaCl*_2_	0.1	*CaCl*_2_	0.1
		*NH*_4_ *Cl*	2	*NH*_4_ *Cl*	2
				*ZnSO*_4_ *7H*_2_ *O*	0.05

All experimental cultures of *R*. *mucilaginosa* UANL-001L were grown in 500 mL Erlenmeyer flasks, containing 200 mL of media. Afterwards, all additional experiments were performed, unless otherwise stated, in 500 mL Erlenmeyer flasks with 200 mL of YMMZ. All cultures were inoculated with a 2 mL aliquot obtained from an overnight yeast culture at an OD_600nm_ of 1. Unless otherwise indicated, all cultures of *R*. *mucilaginosa* UANL-001L were grown at 28 °C and 200 rpm for 96 h.

In this study different bacterial strains were used: *E*. *coli* ATCC 11229, *Staphylococcus aureus* ATCC 6538 and *Pseudomonas aeruginosa* ATCC 27853. These were conserved in LB broth media (Difco, BD) supplemented with 20%(v/v) glycerol. Unless otherwise indicated all of the experimental bacterial cultures were grown in LB broth at 37 °C and 150 rpm for 12 hours.

### Growth Conditions of *Rhodotorula mucilaginosa* UANL-001L and *E*. *coli* Co-cultures

For the co-culture experiments, *E*. *coli* ATCC 11229 was grown in the presence of *R*. *mucilaginosa* UANL-001L in 500 mL Erlenmeyer flasks, containing 200 mL of YM (Yeast Mold) media with different concentrations of added glucose (5, 10 and 15 g/L). The flasks were inoculated with an overnight culture of each of the strains *(E*. *coli and R*. *mucilaginosa)* and they were grown for 72 h at 28 °C and 140 rpms. The inoculum was set to include 1 × 10^6^ CFU/mL of *R*. *mucilaginosa* and a variable inoculum of *E*. *coli* at three different initial concentrations (1 × 10^6^, 3 × 10^6^ and 5 × 10^6^ CFU/mL). After 72 h the biomass from the co-culture was separated from supernatant and the EPS in the supernatant was collected. The extraction and purification of the EPS is thoroughly described in the next section.

### Separation and Purification of EPS

In order to produce exopolysaccharide, the production media broth (200 mL) was inoculated with 1% of an overnight culture (OD_600_ = 1) of the *Rhodotorula mucilaginosa* strain (UANL-001L) and grown at 28 °C and 200 rpm for 96 h. Once the yeast culture reached exponential phase, the biomass was separated from the supernatant by centrifuging the culture at 10,000 rpm for 20 min. Next, the supernatant (containing the EPS) was filtrated through a 0.45 μm pore diameter membrane filter (Milipore). Then, 96% ethanol was added to the filtered supernatant in a 3:1 volume ratio to precipitate the EPS contained in the filtered supernatant sample. The ethanol/supernatant mixture was maintained at 4 °C for 12 hours. Subsequently, the mixture was centrifuged at 10,000 rpm for 20 minutes and the pellets (EPS) were separated from the liquid phase. The precipitated EPS were washed twice with 70% ethanol and, at each step the EPS were centrifuged at 10,000 rpm for 10 min. The recovered pellet was dissolved in deionized water and dialyzed using Spectra/Por molecular porous tubular dialysis membranes for 48 h. The final pellet was then freeze-dried overnight in a lyophilizer (Labconco Freezone-6 model). Finally, the EPS production yield was quantified gravimetrically. All experiments were run in triplicates.

### Cell growth and EPS Kinetic Production

To study the relationship between cell growth and EPS production, *Rhodotorula mucilaginosa* UANL-001L cultures were adjusted to an initial cell density of 0.02 (OD_600nm_) and grown at 28 °C and 250 rpm for 100 hours.

To measure cell growth, aliquots were taken at intervals of 12 hours to monitor OD_600nm_ and CFUs/mL using a platting method. To determine EPS production yield, samples were taken every 24 hours. EPS production yield was determined by precipitation and purification as previously described.

### Scanning Electron Microscopy Analysis

Scanning Electron Microscopy was performed to study the surface morphology and porosity of the exopolysaccharide produced by *Rhodotorula mucilaginosa* UANL-001L. Micrographs were taken using a Nova NanoSEM 200 FEI scanning electron microscope with field emission.

### FT-IR analysis

In order to identify functional groups in the exopolysaccharide produced by *Rhodotorula mucilaginosa* UANL-001L, an FT-IR spectrum of the EPS was performed using a Tensor 27 spectrometer (Bruker, Germany) in the region of 400–4000 cm^−1^.

### Chemical Characterization of EPS

Total amount of carbohydrates present in the EPS samples was determined using the Acid-Phenol Dubois Method^26^. A carbohydrate calibration curve was constructed using glucose as the reference carbohydrate. The solution containing EPS was prepared by dissolving 10 mg of dried EPS in 10 mL of distilled water. A 1 mL aliquot was diluted with 1 mL of 80% (w/v) phenol in water and 5 mL of concentrated H_2_SO_4_. The solution was then heated at 100 °C for 7 minutes. The sample was left at room temperature for 5 hours in order to cool and an aliquot was diluted 1:100 in concentrated H_2_SO_4_ to measure its absorbance at 490 nm using a Varian Cary 50 UV-Vis Spectrophotometer. All experiments were done in triplicates.

Detection of carbonyl groups in the EPS was performed by running a test with 2,4-Dinitrophenylhydrazine (DNPH, Brady’s reagent)^27^. 10 mg of dried EPS were dissolved in 10 mL of deionized water. Then, two drops of this sample, 2 mL of 95% ethanol and 3 mL of Brady´s agent were added to a glass tube and all components were well mixed through vigorous agitation. The presence of a carbonyl group was determined by visually checking if a precipitate was formed in the reaction (positive test)^27^.

Moreover, a qualitative elemental analysis was performed to detect chemical groups containing nitrogen in the EPS samples. 10 mg of the dried EPS were dissolved in 10 mL of deionized water. Two drops of a Fe(NH_4_)_2_(SO_4_)_2_ saturated solution, and 2 drops of 30% KF solution were added to a 1 mL glass tube and mixed well. The solution was alkalized to a pH 9 using concentrated NaOH, and it was then slightly heated in a water bath at 60 °C for 10 seconds to finally be filtered using filter paper under vacuum. Two drops of an FeCl_3_ saturated solution and enough H_2_SO_4_ was added to dissolve the Fe(OH)3 and the solution was acidified. Finally the sample was boiled for 30 seconds and then added aliquots of 10% H_2_SO_4_ until a greenish-blue color appeared, indicating the presence of nitrogen^28^.

Finally, elemental analysis to quantify carbon, nitrogen, hydrogen, and sulfur contents was performed using a Perkin Elmer 2400 Series II CHNS/O Elemental Analyzer. The reported elemental analysis is an average of the results from three EPS samples. The respective standard deviation is also reported.

### Size Exclusion Chromatography

Size exclusion chromatography of EPS was performed using an Äkta Prime Plus FPLC system (GE Healthcare) with a Superdex 200 Increase 10/300 GL column (GE Healthcare). The column was equilibrated with 50 mM TRIS, 100 mM KCl, pH 7.5 buffer pre-filtered through a 0.22 μm filter (Millipore) and a flow rate of 0.3 mL/min. Molecular size calibration was carried out using the gel filtration markers kit (Sigma Aldrich) including: cytochrome C (12.4 kDa), carbonic anhydrase from bovine erythrocytes (29 kDa), albumin bovine serum (66 kDa), alcohol dehydrogenase from yeast (150 kDa), amylase from sweet potato (200 kDa) and blue dextran (>2,00 kDa) under the same conditions. The purified EPS were dissolved in the same buffer and were run after calibration with the standards under the same conditions.

### Monosaccharide composition analysis

#### HPAEC-PAD

1 nmole each in 100 µL was injected on a Dionex ICS-3000, HPAEC-PAD instrument. A Carbopac PA-1 column (4 × 250 mm) was used for profiling of the monosaccharides using a NaOH/NaOAc gradient solvent mixture. Acidic sugars generally elute after 30 min. The EPS samples were hydrolyzed using 2 N TFA at 100 °C for 4 h, followed by removal of excess acid using dry nitrogen flush and then dissolved in water and a known amount was injected on HPAEC-PAD.

### GC-MS analysis

The presence of each of the monosugars was confirmed by matching with the retention time of known monosaccharide standards and the GCMS spectrum. The EPS samples were methanolyzed using 1 M Methanolic-HCl at 80 °C for 16 h, followed by re-N-acetylation and TMS derivative of monosaccharides. Finally, the derivative monosaccharide was dissolved in hexane and injected on the GCMS, equipped with a Restek 5-ms capillary column. Helium was used as the carrier gas. Myo-inositol was used as an internal standard, only the detected monosaccharides are labeled.

### Antibiofilm Activity of EPS

The antibiofilm assays were performed by first resuspending the lyophilized EPS in bidistilled water to produce a 400 mg/mL stock solution. The EPS stock solution was stored at 4 °C.

Antibiofilm activity of the EPS was analyzed by an adapted microtiter biofilm formation assay. Aliquots of each of the different bacteria cultures tested in this work were transferred into the 96-well microtiter plate obtaining a final dilution of 1:200. Afterwards, different concentrations of EPS were added to each well as follows: 2500 ppm, 2000 ppm, 1000 ppm, 500 ppm, 250 ppm, 125 ppm and 75 ppm. A control culture was grown in parallel without having added EPS. All biofilm cultures were incubated at 37 °C for 36 hours under static conditions.

Biofilm inhibition was measured using the crystal violet staining method^29^. In order to discard planktonic cells from biofilm cultures, after the incubation period the supernatant was removed and each of the microtiter plates were washed 3 times with bidestilled water. The plates were then dried with hot air and the biofilm that remained in each well of the titer plates was stained with 250 µL of 1% crystal violet dye for 10 minutes. After 10 minutes, the dye was washed away with bidestilled water. Biofilms were then de-stained by adding 250 µL of an ethanol 96% solution. The ethanol/crystal violet dye solution at each of the wells is finally transferred to another microliter plate were the optical density is measured at 590 nM using a Varioskan (Thermo Scientific, Burlington, ON, Canada) spectrophotometer. The values are given as the mean of 6 biological replicates and their respective standard deviations are reported.

### Bacterial Growth Inhibition with EPS

For the antimicrobial assays, the lyophilized EPS were resuspended in bidistilled water to produce a 400 mg/mL stock solution that was stored at 4 °C. The effect of EPS on bacterial growth and its antibacterial activity was assayed by the microdilution method. Suspensions of each of the different bacteria cultures tested were inoculated into a 96-well microtiter plate (10^7^ cells/well) and incubated in the presence of a range of different EPS concentrations as follows: 2500 ppm, 2000 ppm, 1000 ppm, 500 ppm, 250 ppm, 125 ppm and 75 ppm. As a control, a culture was grown in parallel without the addition of EPS. All of the tested wells contained a final volume of 200 µL per well. All of the cultures were grown at 37 °C and 150 rpm for 18 hours. Finally, bacterial growth inhibition was quantified by reading the OD of the treatments at 600 nm. The values are given as the mean of 6 biological replicates and their respective standard deviations are reported.

### Statistical analysis of Antimicrobial and Antibiofilm Analysis

Statistical analysis was performed using GraphPad Prism version 7.0 (GraphPad Software, San Diego, CA, USA). One way Analysis of Variance (ANOVA), followed by Dunnett’s or Tukey´s post hoc tests, when appropriate, were used to determine significant differences (P-value < 0.05) between treatments in the antimicrobial and antibiofilm assays. All experiments were performed in biological triplicates and the mean is reported with the corresponding standard deviations.

### Fluorescence Microscopy of Microbial Cells

10^5^ bacterial cells were treated with 0, and 2500 ppm of EPS for 20 hours at 37 °C and 150 rpm. After treatment, cells were stained with 5 µg/mL of Propidium Iodide (PI) for 1 hour at 37 °C-150 rpm. Then, 20 µL of the control and treated stained cells were spread in a glass slide, previously cleaned with 96% ethanol. The sample was allowed to air dry and the slides were then fixed by adding 200 µL of absolute methanol for 2 minutes. Methanol excess was decanted and slides were washed twice with PBS. Images were acquired with a LEICADM 3000 using the 100X objective with a Filter system Y3 ET.

### Citotoxicity of Exopolysaccharide

#### Cell culture

H9c2 cells (ATCC, Manassas, VA, USA) were plated in 12-well trays at a concentration of 2 × 10^4^ cells per well in DMEM media supplemented with 10% fetal bovine serum (FSB), penicillin (100 U/mL) and streptomycin (100 µg/mL). After reaching 70–80% confluence the cells were treated with different concentrations 5,000 ppm, 2500 ppm, 2000 ppm, 1000 ppm, 500 ppm, 250 ppm, 125 ppm and 75 ppm of EPS for 24 h at 37 °C in an atmosphere. composed of 95% air and 5% CO_2_. A culture with no EPS added was grown in parallel as a control.

#### Assessment of apoptosis and necrosis by flow cytometry

H9c2 cells plated in 12-well trays at a concentration of 2 × 10^4^ cells per well in 1 mL DMEM media, were treated with 5,000 ppm, 2500 ppm, 2000 ppm, 1000 ppm, 500 ppm, 250 ppm, 125 ppm and 75 ppm of EPS. After 24hr of incubation the cells were trypsinized and harvested. Approximately 8 × 10^4^ cells/well were washed with media supplemented with FBS to neutralize the trypsin and centrifuged at 1,400 rpm for 7 min, at room temperature. After washing the cells 2 times in media, the cells were resuspended in a Tyrode solution in Falcon FACS tubes (Corning, Life Sciences, Mexico).

To assess apoptosis, an Annexin V Apoptosis Detection kit conjugated with PE-Cy7 (eBioscience, San Diego, CA, USA) was used. Next, the cells were resuspended in Tyrode solution plus 2.5 mM CaCl_2_ and stained with Annexin V. Cells were incubated for 10 minutes at room temperature in the dark. After incubation, a Tyrode plus 2.5 mM CaCl_2_ solution was added and the cells were washed at 1,400 RPM for 7 min at room temperature. After washing, the cells were resuspended in Tyrode plus 2.5 mM CaCl_2_ solution. To assess viability, propidium iodide was added to discard necrotic cells. For each tube 20,000 viable events were recorded. Samples were analyzed with a FACSCanto II (Becton Dickinson, San Jose CA) cytometer with a 488 nm and a 633 nm laser. To analyze the proportions between viable and apoptotic cells, software compensation was performed using Flowjo VX (Treestar, Oregon, USA). After discarding doublets, Annexin V positive cells were deemed as apoptotic, propidium iodide positive cells were deemed as necrotic, while cells without any staining were deemed as viable cells. As positive controls for apoptosis Staurosporin was used at a concentration of 20 µM. For necrosis Doxorubicin was used as a control at 30 µM.

All experiments were performed in biological triplicates and the statistical data are presented as mean ± standard deviation. Fluorescence values represent the mean of the Median Fluorescence Intensity of each individual assessment. Comparison between samples were performed by Mann Whitney U test or Kruskal-Wallis for three or more samples, followed by Dunnett´s, Tukey´s or Bonferroni´s *post hoc* tests, when appropriate, to compare experimental groups. Differences were considered significant when p < *0*.*05*. Data processing, graphs and statistical analysis were performed with GraphPad Prism (V.5.01; La Jolla, CA, USA) and OriginPro 8.1 SR3 v8.1 (OriginLab Corporation, Northampton MA, USA).

#### In Vivo Murine Toxicity Experiments

All the experiments were performed in accordance to the animal care guidelines of the Guide for the Care and Use of Laboratory Animals published by the US National Institutes of Health (NIH Publication No. 85–23, revised 1996). The animal use and care committee of the Tecnologico de Monterrey-Medical School approved all procedures. Female C57BL/6 mice (6–8 weeks of age) received a single dose of EPS (2000 mg/Kg of body weight) by gavage^30^. The maximum dose was chosen based on the OECD 423 protocol^31^. The animals were weighed daily during a week. Mice were examined for behavioral changes or any clinical sign of toxicity every hour during the first 8 h following the treatment and thereafter once a day for a week. Later, the mice were euthanized with a lethal dose of sodium thiopental administered intraperitoneally. After euthanasia, all animals were submitted to necropsy and organs were macroscopically inspected for any abnormality. Hematocrit, leucocytes and erythrocytes were measured and compared with untreated mice.

## Results and Discussion

### Kinetics of Cellular Growth and EPS production

**Figure 1:**
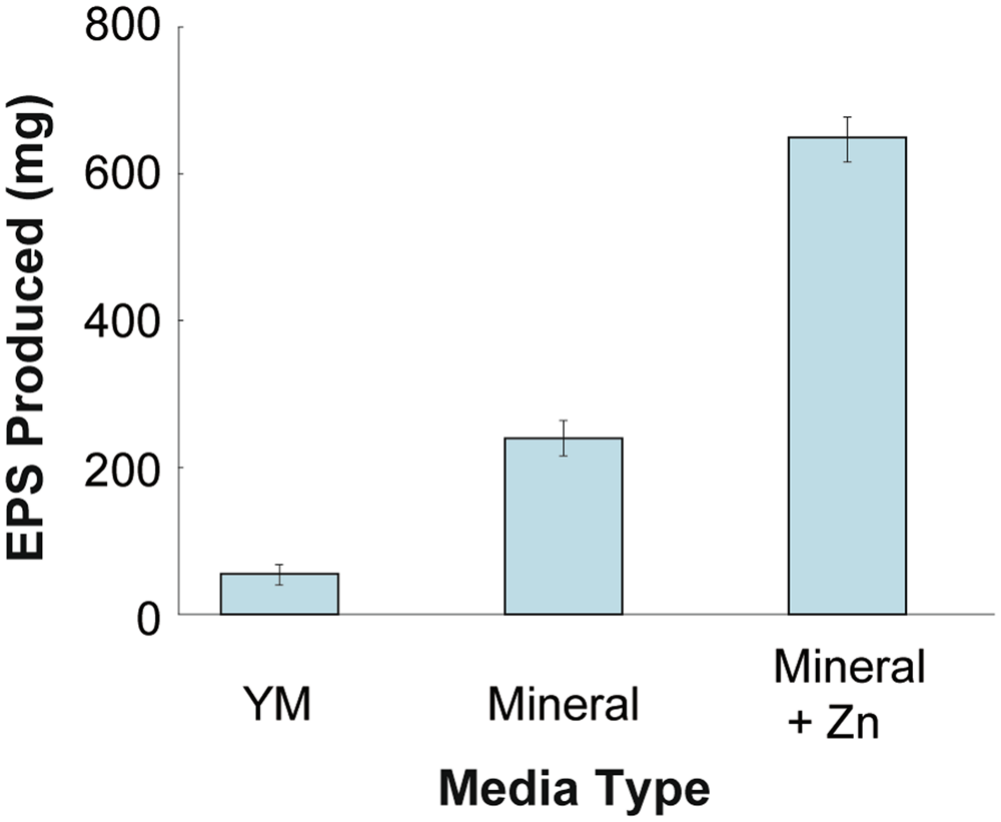
EPS production in different media formulations. EPS production at 72 h, when *R*. *mucilaginosa* UANL-001L is grown in YM media, Mineral Media and Mineral Media + Zn. Mean + − SD, n = 3 error bars are reported.

Three different growth media were explored in order to determine the optimal components that stimulated EPS biosynthesis in *R*. *mucilaginosa* UANL-001L. Our research group previously found that *R mucilaginosa* UANL-001L increases EPS biosynthesis in the presence of different heavy metals^25^. Therefore, the difference in composition between the YM media and the other two media tested was the addition of Zn and other transition metals (K, Na, Mg and Ca). As can be observed from Fig. 1, EPS biosynthesis is increased when transition metals are added to the media. When compared with EPS produced by *R*. *mucilaginosa* grown in YM alone, EPS biosynthesis is increased by 900%, when *R*. *mucilaginosa* is grown in YM media with added K, Na, Mg and Ca, and 3000% when it is grown in YM media with added K, Na, Mg. Ca and Zn.

**Figure 2:**
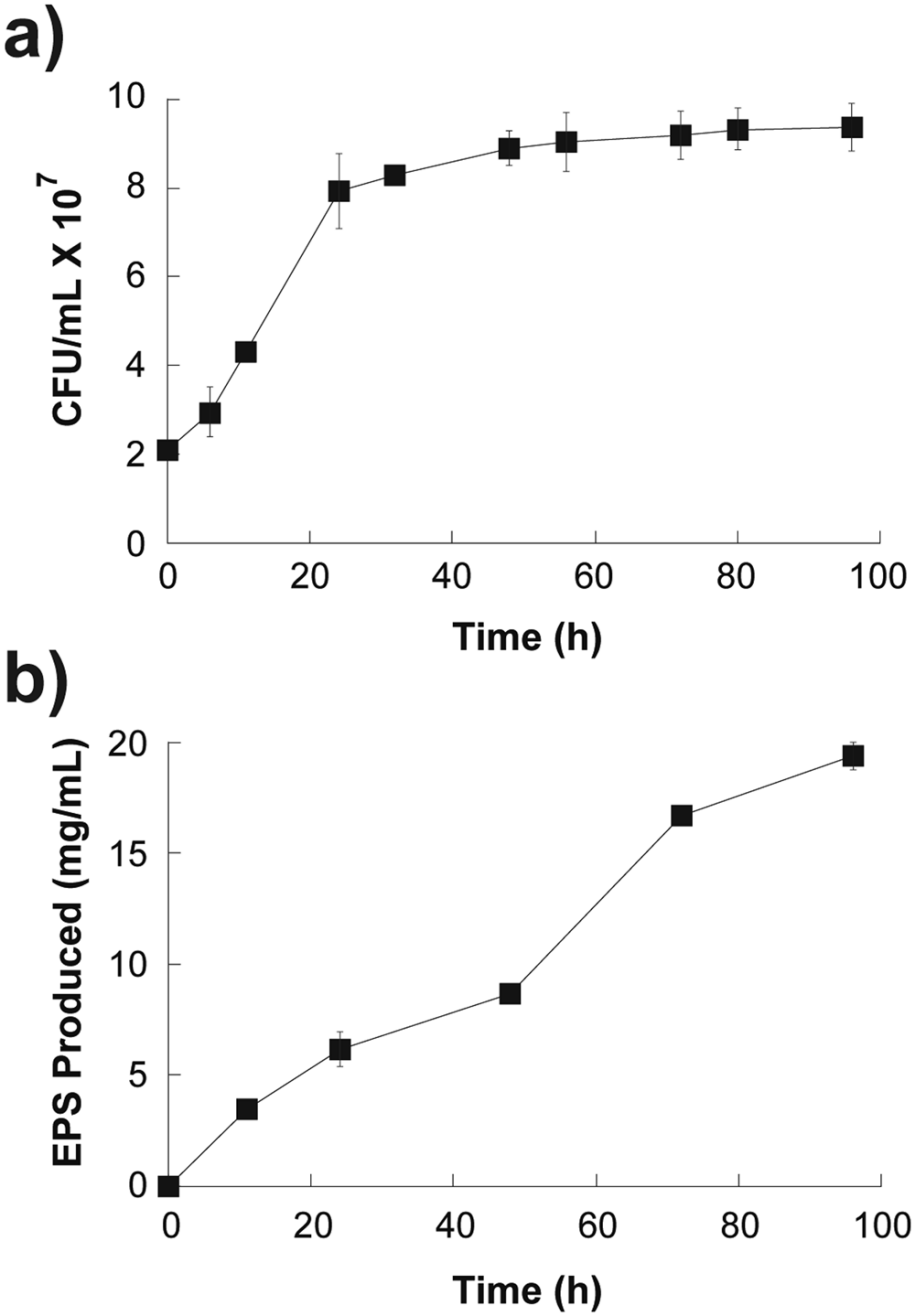
EPS biosynthesis as a function of cellular growth. (**A**) Cell growth kinetics for 96 h. (**B**) EPS production kinetics for 96 h. Mean + − SD, n = 3 error bars are reported.

EPS biosynthesis in *R mucilaginosa* UANL-001L was further studied in the best performing medium (YM-Mineral-Zn) as a function of cellular growth. Under experimental conditions, stationary phase was reached at 48 hours with an estimate of 8.2 × 10^7^ CFU/mL (Fig. 2A). Exopolysaccharide biosynthesis began at exponential phase at 12 hours, reaching the highest EPS production yield (19 mg/mL) at 96 hours (Fig. 2B). These results correlate with the data obtained recently by our research group^25^.

The results show that the exopolysaccharide is mainly produced when the microbial culture is in the stationary phase. It might be assumed that when the stationary phase is reached, the metabolic pathway changes to EPS production, instead of cellular growth, as has been described by other authors^32,33^.

### EPS Production in a *Rhodotorula mucilaginosa* UANL-001L – *E*. *coli* coculture

**Figure 3:**
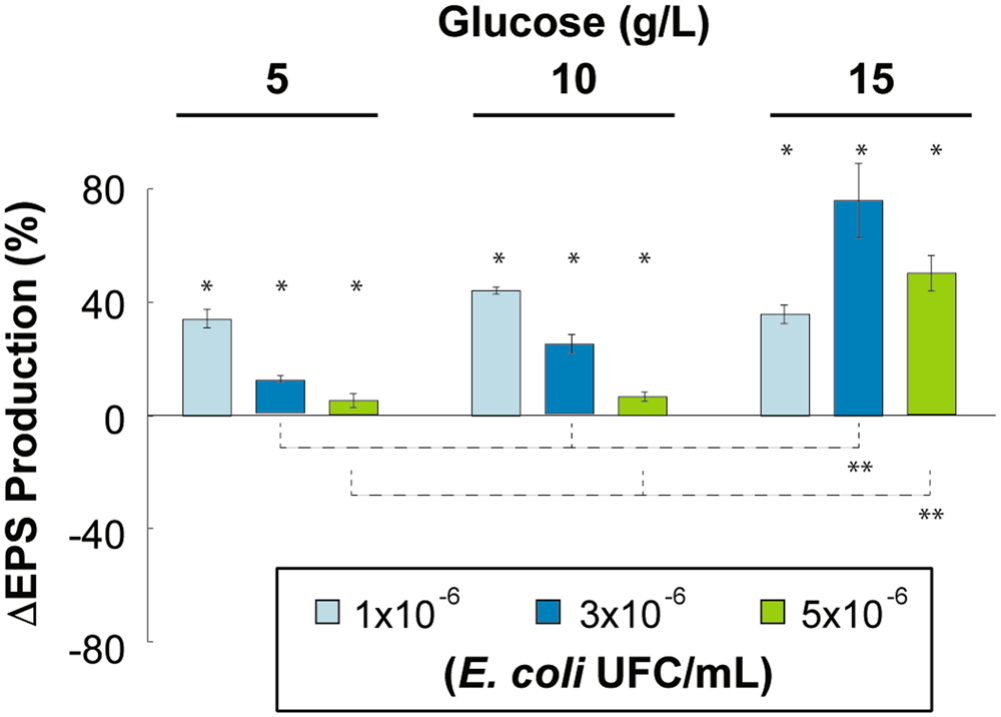
EPS biosynthesis in *R*. *mucilaginosa* UANL-001L when co-cultured at low, medium and high levels of *E*. *coli* initial inoculum and glucose concentration in the media. EPS production after 72 h of co-culture is reported as a percentage increase compared to EPS production when *R*. *mucilaginosa* UANL-001L is grown alone. Mean + − SD, n = 3 error bars are reported. A (*) above the bar represents a P < 0.05 between groups at same glucose concentration. A (**) above the bars represent a P < 0.05 between groups at same initial *E*. *coli* inoculum.

In order to explore EPS production by *R*. *mucilaginosa* UANL-001L in the presence of another microorganism; a set of experiments were performed where the *R*. *mucilaginosa* initial inoculum concentration was maintained constant and glucose and the initial inoculum of *E*. *coli* were varied between 3 levels (low, medium and high). As can be observed from Fig. 3, in general, EPS production in the cocultures is increased between 10 and 80%, when compared to EPS production when *R*. *mucilaginosa* UANL-001L is grown alone. Moreover, for each of the initial *E*. *coli* inoculum experiments, doubling glucose concentrations from 5 to 10 g/L is not sufficient to increase EPS production (Fig. 3). This is a common phenomenon since it has been previously reported that metabolite biosynthesis does not correlate linearly with the amount of carbon sources in the media^34,35^. It can be suggested that at these coculture conditions the carbon source is still limited and therefore the EPS production is not increased. However, when glucose concentration is raised to 15 g/L, the carbon source is less limiting, especially for the 3 × 10^6^ *E*. *coli* initial inoculum, and the *E*. *coli* initial concentration plays a different effect, triggering a 2.5 fold increase in EPS production. For the low 1 × 10^6^ initial inoculum concentration, even though EPS production is increased by almost 40% compared to EPS production in *R*. *mucilaginosa* grown alone, the results also show that production is kept almost constant, independent of increases in the amount of glucose added (Fig. 3). Together, these results show that, like in other coculture systems reported in the literature, EPS production depends on the interaction between carbon source availability and the initial size of the *E*. *coli* inoculum^36^. These results also suggest that EPS production in *R*. *mucilaginosa* UANL-001L is a mechanism of defense triggered by the presence of a competing bacterial species; a phenomenon that has been observed in both bacterial and fungi species.

### Chemical Characterization of the EPS using FT-IR

**Figure 4:**
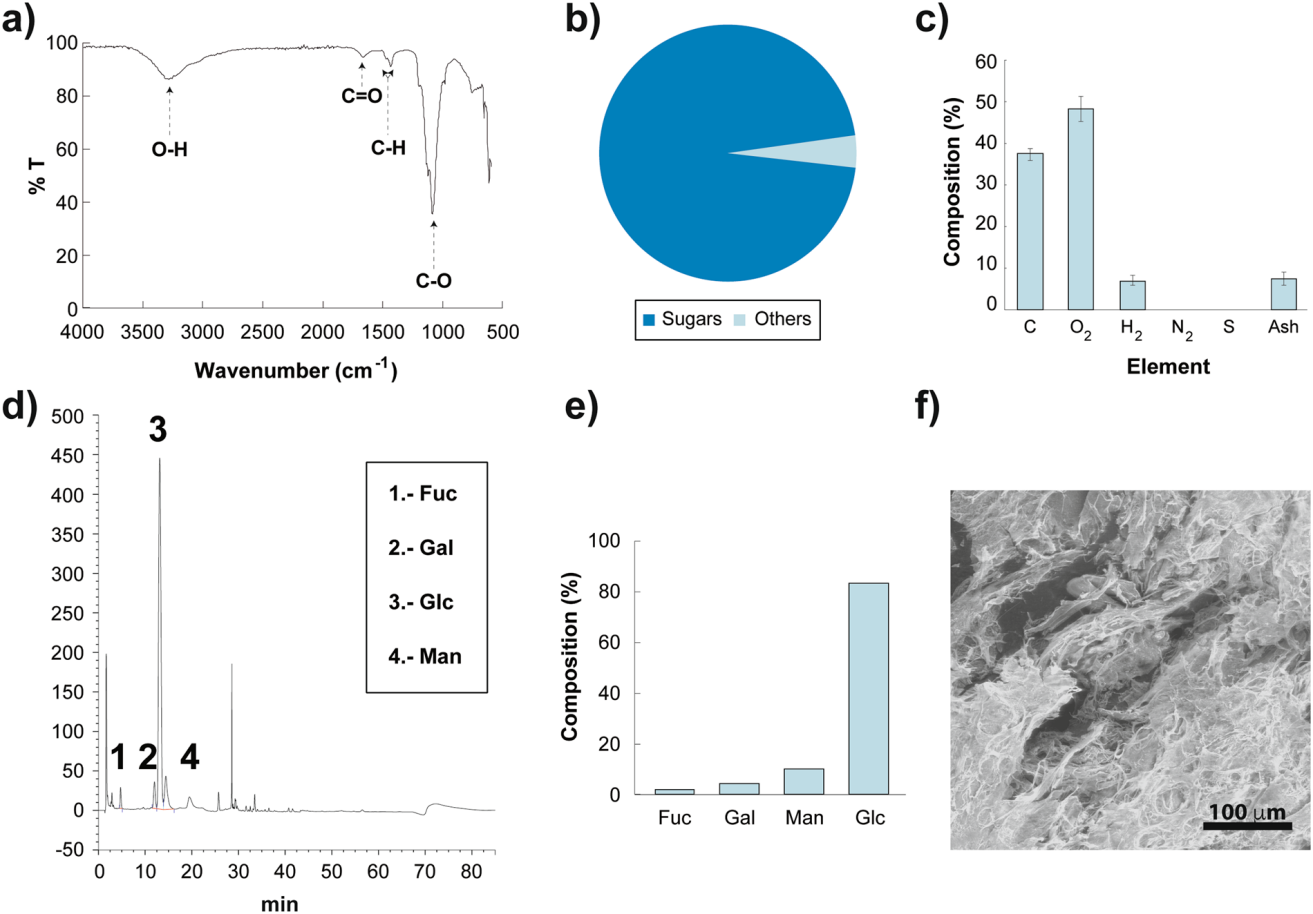
Chemical and Physical Properties of the EPS biosynthesized by *R*. *mucilaginosa* UANL-001L. The data show chemical and physical analysis of the EPS produced by *Rhodotorula mucilaginosa* UANL-001L. (**a**) FTIR of the EPS with the different peaks highlighted and tagged with the chemical group corresponding to each specific wavenumber. (**b**) Percentage of carbohydrates present in the EPS. (**c**) C, O, H, N, S and ashes percentage composition of the exopolysaccharides. (**d**) GC-MS spectrum of EPS produced by *R*. *mucilaginosa* UANL-001L, with all of the monosugars detected labeled. (**e**) Composition of each of the monosugars present in the EPS. (**f**) SEM image showing morphology of EPS.

Chemical characterization of the biosynthesized exopolysaccharide was first performed through FT-IR. The spectrum is shown in Fig. 4a where different regions of interest are observed. The peak at 3600–3200 cm^−1^ was assigned to hydroxyl groups from polysaccharide^37^. The weak peak at approximately 2920 cm^−1^ corresponds to CH_2_, a band at 1650–1540 cm^−1^ corresponds usually to enol and amide groups^19^. The absorption peak at approximately 1365 cm^−1^ corresponds to carboxyl groups^38^. Finally, the broad stretch region from 1000–1200 cm^−1^ corresponds to the C-O, C-C stretching, C-O-C and C-O-H deformation vibrations of polysaccharides. The strongest peak at 1084 cm^−1^ was an indicative that the sample is a polysaccharide^39^.

Furthermore, to decipher the chemical groups contributing to the 1649 cm^−1^ wavelength, a chemical assay using 2,4-dinitrofenilhydrazone was performed to detect the presence of carbonyl (C = O) groups in the samples. The assay resulted positive for the presence of carbonyl groups in the EPS. Next, through an initial qualitative elemental analysis, amino and amido groups were discarted due to the absence of nitrogen in the samples. Together, these chemical assays show that the absorption band at 1649 cm^−1^ corresponds to the C=O stretching vibration in the carbonyl groups.

Total amount of carbohydrates in the EPS was quantified through the Debois method. The total percentage of carbohydrates in the samples ranged from 91 to 96% with variations of only 5% (Fig. 4b). Furthermore, quantitative data was obtained using an Elemental Analyzer. As shown in Fig. 4c, carbon, hydrogen and oxygen made up for 93% of the total samples. The results again demonstrate the absence of N and S in the samples; confirming that amino and amido groups were absent in the EPS produced (Fig. 4c).

### Monosaccharide composition analysis

The EPS samples were further characterized through an HPAEC-PAD analysis. Figure 4d, shows that the EPS are composed of the following monosaccharides: fucose, galactose, mannose and glucose. The most abundant monosaccharide in the composition of the EPS is glucose with 82%, followed by mannose, galactose and fucose, with the least contribution to the composition (Fig. 4e). After hydrolysis there were no uronic acids in the sample, suggesting that the multiple peaks of glucose shown in the GC-MS spectrum, correspond to intact ring forms of sugars present in different configurations^40^. Subsequently, the presence of these sugars was confirmed by matching the retention time of known monosaccharide standards with the GC-MS spectrum. The composition of the exopolysaccharide reported in this work indicates that it is a novel biopolymer produced by the *R*. *murcilaginosa* UANL-001L strain. The exopolysaccharide here reported resembles the composition of other microbial EPS that have glucose as their main monosaccharide^41^. Moreover, its composition is very similar to a mannose-rich acidic heteropolysaccharide, produced by *R*. *glutinis* KCTC 7989, and composed of 85% of neutral sugars (fucose, mannose, galactose, and glucose) and 15% uronic acid^33,42^.

### Physical Properties of EPS using SEM

Through size exclusion chromatography, the molecular weight of the EPS was found to be 18.85 kDa, considered a low molecular weight biopolymer compared to EPS produced by other microorganisms^43^. The EPS morphology was analyzed using an SEM since it is a technique that can be used to provide insights of physical properties^44^. The micrographs of the EPS from *R*. *mucilaginosa* UANL-001L show that the EPS exhibit a compact and granular surface (Fig. 4f), suggesting that these polysaccharides might have gelling and emulsifying properties^39,44^. Due to the aforementioned characteristics, this polysaccharide could be applied as a thickener, stabilizer and as an emulsifier agent in the food and cosmetic industry. Moreover, this polymer could be used to develop encapsulation technologies for the delivery of drugs or bioactive compounds^45^.

### Antibiofilm activity

Antibiofilm activity of EPS was analyzed against one Gram positive (*S*. *aureus* ATCC 6538) and two Gram negative (*E*. *coli* ATCC 11229 and *P*. *aeruginosa* ATCC 27853) strains, in order to determine the antimicrobial spectrum of the exopolysaccharide.

**Figure 5:**
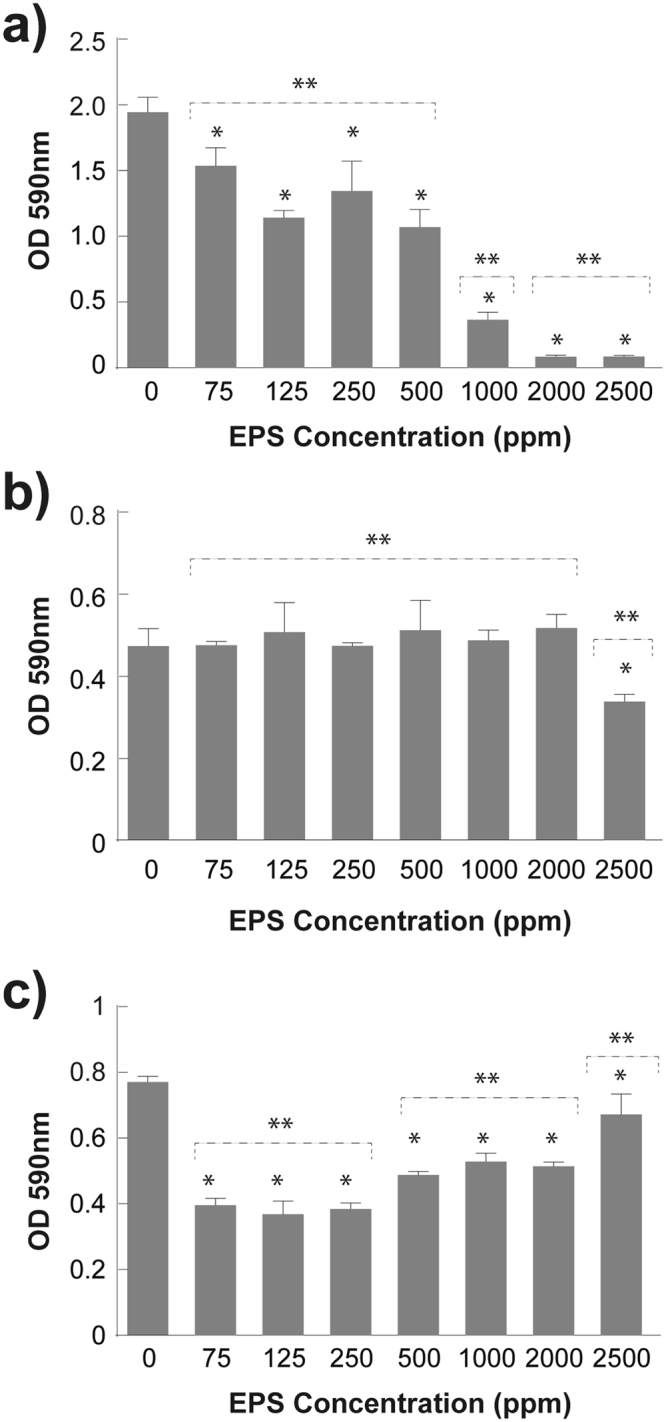
Antibiofilm Activity of the EPS biosynthesized by *R*. *mucilaginosa* UANL-001L. Biofilm formation is measured after the treatment of bacterial cultures with a range of EPS concentrations. The results correspond to (**a**) *S*. *aureus* ATCC 6538, (**b**) *P*. *aeruginosa* ATCC 27853 and (**c**) *E*.*coli* ATCC 11229. Mean + − SD, n = 3 error bars are reported. A (*) above the bar represents a P < 0.05 between treatments and the control. A (**) above the bars represent a P < 0.05 between groups of treatments.

The effect of EPS on biofilm formation is reported in Fig. 5. EPS presents a dose-dependent inhibitory effect *on S*. *aureus* biofilm formation, inhibiting 81% of biofilm formation when a concentration of 1000 ppm was used (Fig. 5a). When higher concentrations of EPS (2000 ppm and 2500 ppm) were used, further inhibition (96%) was shown (Fig. 5a). EPS from *Rhodotorula mucilaginosa* UANL-001L exhibits a higher antibiofilm activity at minor concentrations compared to EPS from *Lactobacillus plantarum YW32* (5 mg/mL) and *Streptococcus phocae PI80* (5 mg/mL), which inhibited biofilm formation by 45% and 51%, respectively^17,46^.

The addition of *R*. *mucilaginosa* EPS at 2500 ppm was able to inhibit 30% of *P*. *aeruginosa* biofilm formation (Fig. 5b). Similar to the *R*. *mucilaginosa* EPS activity, r-EPS (1 mg/mL) from *Lactobacillus acidophilus A4*, which was able to reduce biofilm formation by 40%^47^. Such is also the case of EPS (1 mg/mL) from *E*. *faecium MC13*, capable of reducing *P*.*aeruginosa* biofilm by 15%^48^. Moreover, a reduction of 86% on biofilm formation was achieved by a treatment with B4-EPS1 from *Arthrobacter sp*. *B4* at a lower concentration (50 µg/mL)^49^. For the case of biofilm formed by *E*. *coli* (Fig. 5c), the results show that biofilm formation was reduced when lower concentrations of *R*. *mucilaginosa* EPS (250 ppm, 125 ppm and 75 ppm) were added. In contrast, biofilm formation was recovered at higher concentrations (2500 ppm, 2000 ppm, 1000 ppm, 500 ppm). Similar results have been reported before, the capsular polysaccharide of *Vibrio sp*. QY101 stimulated biofilm establishment of *P*. *aeruginosa* ATCC27853, *S*. *aureus* and *E*. *faecalis* OG1RF^50^. Therefore, *Rhodotorula* EPS may induce an unidentified mechanism in *E*.*coli* cells that enables biofilm formation at higher EPS concentrations.

EPS from *R*. *mucilaginosa* exhibited antibiofilm activity against Gram-positive and Gram-negative bacteria. In fact, some authors had reported that exopolysaccharides are able to interfere with cell-surface interaction (initial adhesion of biofilm development) via modification of physicochemical properties of biotic and abiotic surfaces, inhibition of cell to cell interaction and downregulation of biofilm-forming genes^24^.

These EPS antibiofilm properties unblock potential applications in health and industrial settlings, where bacterial cells form biofilms to colonize tissues and medical devices or attach to surfaces affecting industrial process. The EPS produced by *R*. *mucilaginosa* UANL-001L has very interesting applications as an antiadhesive or antibiofouling agent to prevent bacterial biofilm formation.

### Bacterial Growth inhibition

**Figure 6:**
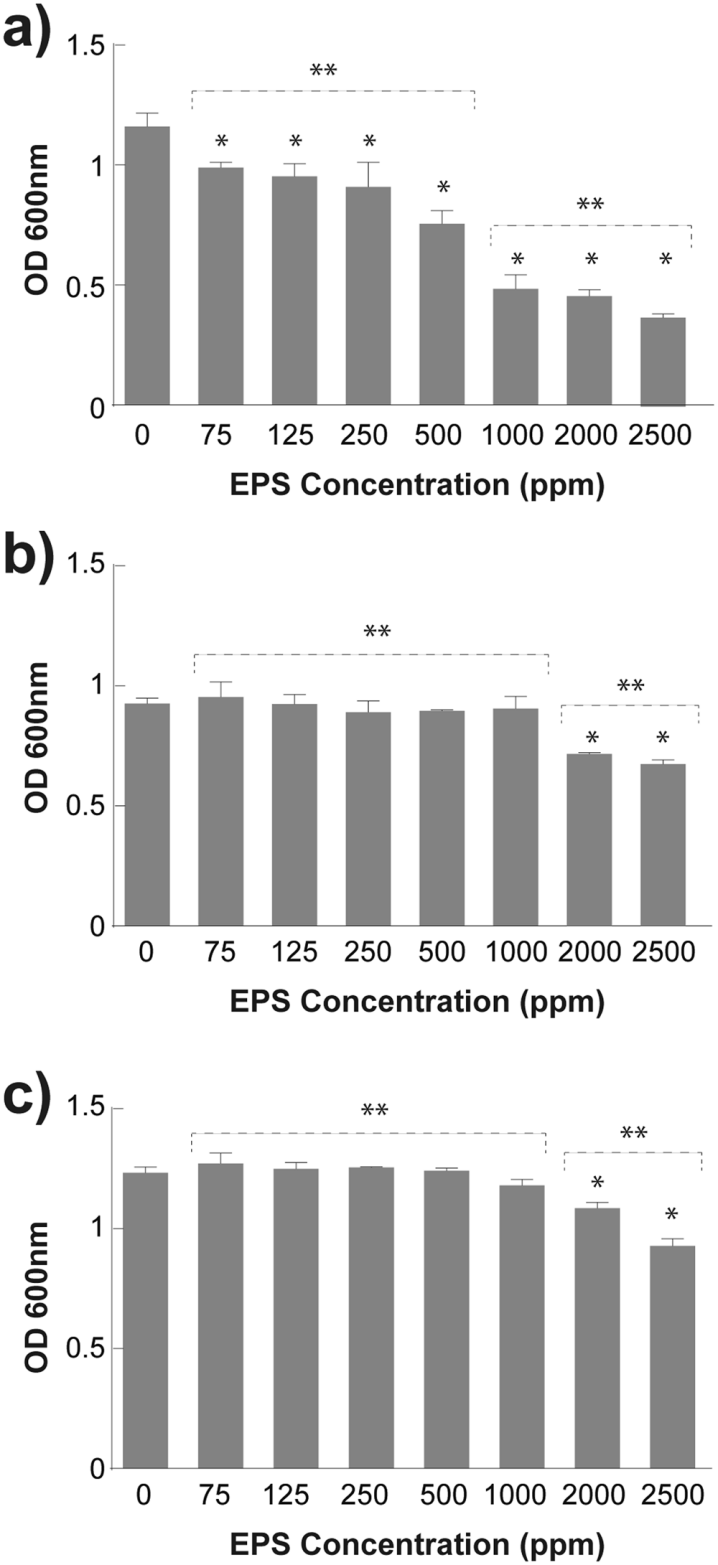
Antimicrobial Activity of the EPS biosynthesized by *R*. *mucilaginosa* UANL-001L. Microbial growth in liquid culture is measured after the treatment of bacterial cultures with a range of EPS concentrations. The results correspond to (**a**) *S*. *aureus* ATCC 6538, (**b**) *P*. *aeruginosa* ATCC 27853 and (**c**) *E*.*coli* ATCC 11229. Mean + − SD, n = 3 error bars are reported. A (*) above the bar represents a P < 0.05 between treatments and the control. A (**) above the bars represent a P < 0.05 between groups of treatments.

EPS antibacterial activity was measured in order to explore its toxicity against both bacterial models. Different EPS concentrations were used against *E*.*coli*, *S*. *aureus* and *P*.*aeruginosa*, displaying diverse effects amongst the tested bacteria. (Fig. 6).

A dose-dependent inhibitory effect was observed when serial concentrations of EPS were used against *S*.*aureus* (Fig. 6a). EPS at concentrations between 1000 and 2500 ppm, showed the strongest antibacterial activity, inhibiting 60% of bacterial growth (Fig. 6a). Similarly, *E*.*coli* (Fig. 6b) and *P*. *aeruginosa* (Fig. 6c) were susceptible to EPS at 2000 and 2500 ppm, inhibiting bacterial growth at 27% and 24%, respectively. Although the exact antibacterial mechanism of the EPS from *Rhodotorula mucilaginosa UANL-001L* remains unclear some authors have suggested that microbial exopolysaccharides could modify and disrupt bacterial cell surface leading to the leakage of intracellular proteins and metabolites, which results in cell death^16,51^. Moreover, based on the literature published, this is the first report on antibiofilm and bactericidal activity exhibited by EPS produced in *R*. *mucilaginosa*.

### Insight into the Antimicrobial Mechanism of Action

**Figure 7:**
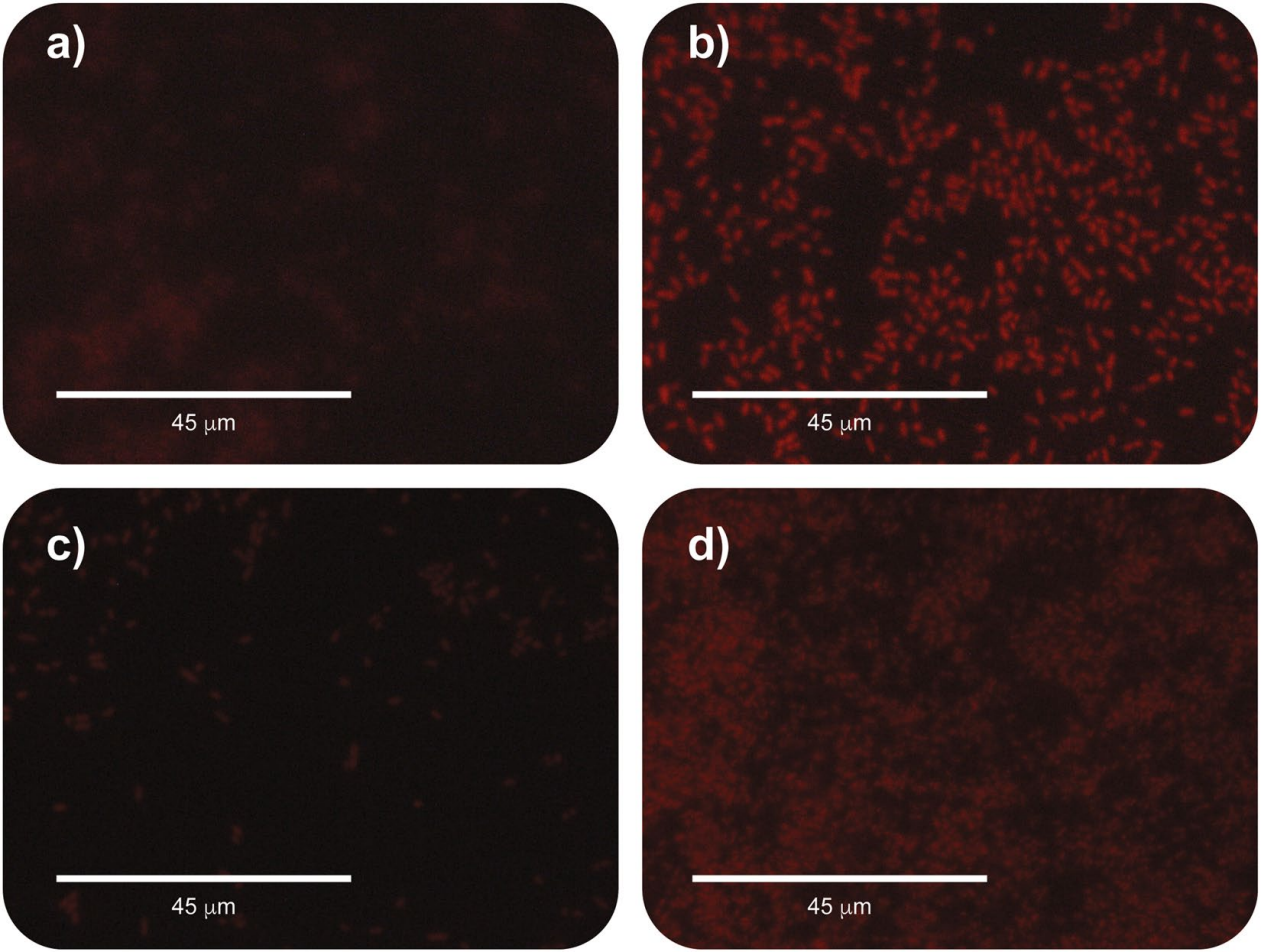
Permeability assay of *E*. *coli* and *P*. *aeruginosa* treated with EPS. Fluorescence micrographs of PI stained bacteria. (**a**) Control untreated *E*. *coli*; (**b**) *E*. *coli* treated with 2500 ppm of EPS; (**c**) Control untreated *P*. *aeruginosa*; (**d**) *P*. *aeruginosa* treated with 2500 ppm of EPS.

An insight into the antimicrobial mechanism of action was sought through the analysis of bacterial membrane disruption, using a fluorescence PI stain^52^, after EPS treatments. As observed in the micrographs, *E*. *coli* (Fig. 7b) and *P*. *aeruginosa* (Fig. 7d) treated with 2500 ppm of EPS show, qualitatively, increased fluorescence compared to the untreated *E*. *coli* (Fig. 7a) and *P*. *aeruginosa* (Fig. 7c). These results, combined with the data on antimicrobial activity, show that even when the exopolysaccharide has a low growth inhibition on the bacterial strains (2500 ppm), the EPS obtained from *R*. *mucilaginosa* disrupts membrane integrity and causes an increase in cell permeability. This can be hypothesized to be at least one of the EPS´s mechanisms of antimicrobial action against competing microorganisms. Moreover, this surface interaction between the *R*. *mucilaginosa* EPS and the membrane of the bacteria, could be one of the antibiofilm mechanisms since the disruptive interaction would act as an anti-adhesive^23^. Antibiofilm exopolysaccharides have been reported to modify the physical properties of biotic and abiotic surfaces^24^. Thus, the antibacterial and antibiofilm effect of the EPS reported in this work may be caused by modification of the *E*. *coli* and *P*. *aeruginosa* cell membrane.

### Exopolysaccharide Cytotoxicity

**Figure 8:**
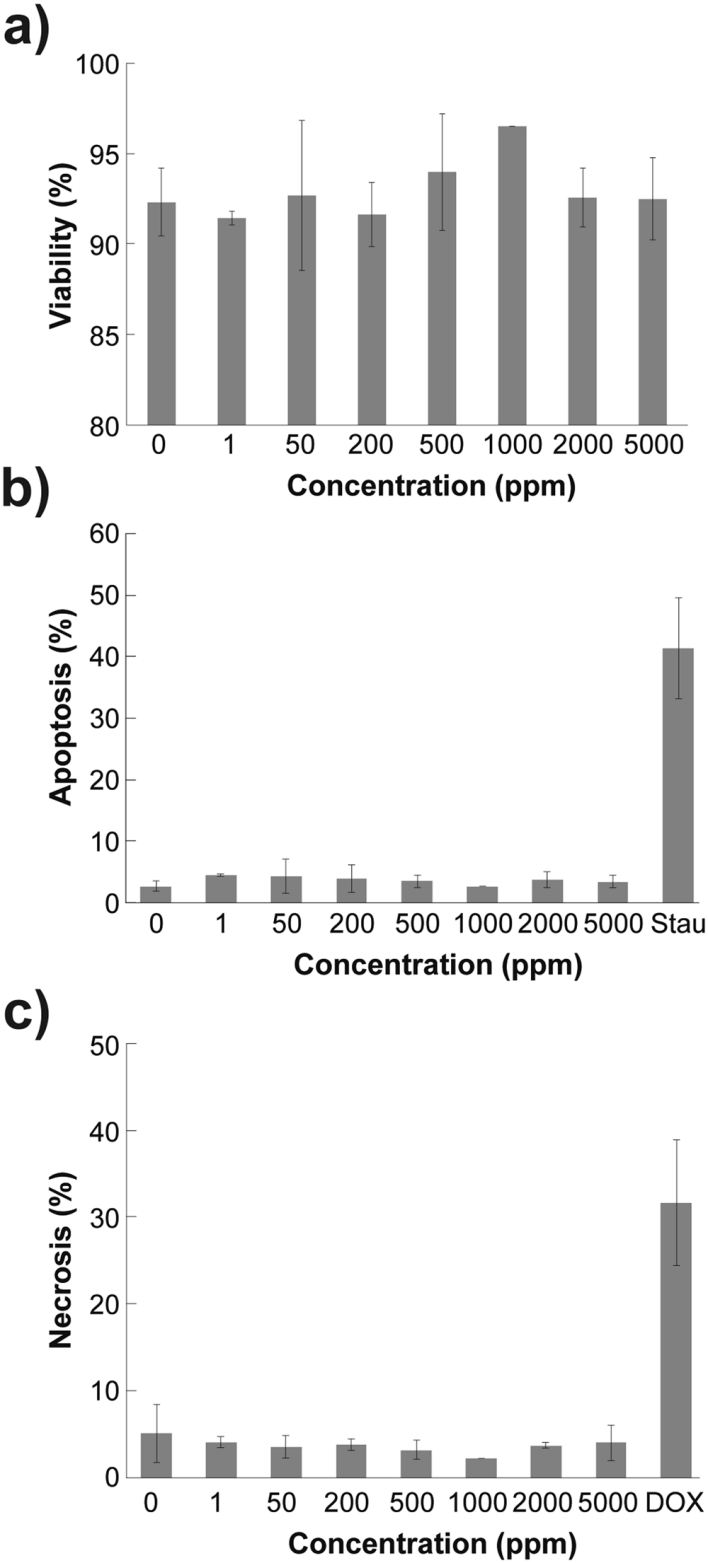
A dose range of 0.1–5000 µg /mL EPS does not induce apoptosis or necrosis after 24 hrs. There were no differences in the percentage of induction of necrosis or apoptotic in a dose dependent setting. (**a**) Viable cells, (**b**) Apoptotic cells and c) Necrotic cells. Results are means ± S.D. n = 3–6, p values for viability p = 0.83, apoptosis p = 0.6 and necrosis p = 0.8.

In order to assess the cytotoxic effects of EPS in a biological/translational setting we tested different concentrations *in-vitro* with a relevant cell line. H9c2 cells are cardiac ventricular myoblasts that resemble the functionality of cardiac myocardiocytes. The results in Fig. 8a show that EPS was not toxic, since viability remained constant above 92%, using doses ranging between 0–5000 µg/mL. There were no statistical differences between each of the doses (p = 0.83). The frequencies of both mean apoptotic (Fig. 8b) and necrotic cells (Fig. 8c) were maintained at values below 5%, with no statistical difference between them (p = 0.6 and p = 0.8, respectively). Moreover, we tested positive controls for apoptosis and necrosis. Cells were treated with Staurosporin, as a positive control for apoptosis, and with Doxorubicin, as a positive control for necrosis. The results show a 40% of apoptosis for the Staurosporin treatment and a 32% of necrotic cells for the Doxorubicin treatment. Together, these results demonstrate that the EPS does not exhibit cytotoxic effects to the tested cell line.

### Exopolysaccharide Cytotoxicity *In Vivo*

**Figure 9:**
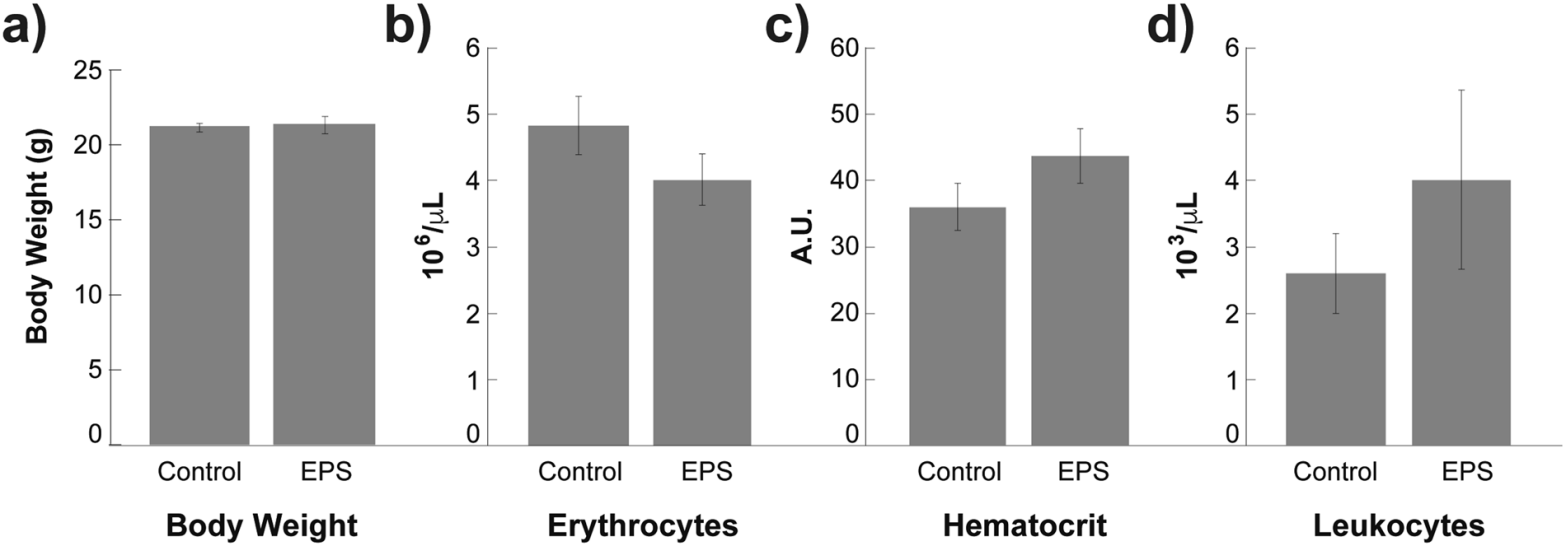
Murine Model Shows No Cytotoxicity of EPS. There were no statistical differences between control and the treated mice in: (**a**) Body Weight (p = 0.82); (**b**) Erythrocytes (p = 0.21); (**c**) Hematocrit (p = 0.23); (**d**) Leukocytes (p = 39) Results are means ± S.D, n = 3.

A murine cytotoxicity model was performed to test effects of EPS from *R*. *mucilaginosa* UANL-001L. No deaths or any other clinical or behavioral signs of toxicity were observed in mice treated with EPS. All EPS-treated mice (2000mg/kg bw po) maintained their body weight throughout 1 week post-treatment. The mean body weight between the untreated and EPS treated mice showed no statistically significant differences (Fig. 9a). Blood tests results show that there were no differences in erythrocyte (Fig. 9b), hematocrit (Fig. 9c) and leukocyte (Fig. 9d) counts between the treated and untreated mice. In addition, no gross pathological abnormality was observed in major organs at necropsy, therefore histology was considered unnecessary. Since this EPS upper limit dose caused no deaths or any other discernible signs of toxicity, lower doses were not tested

## Conclusions

The novel strain *Rhodotorula mucilaginosa* UANL 001 L produces a non-cytotoxic novel exopolysaccharide that presents interesting antibiofilm and antimicrobial properties. This is the first report of an antimicrobial and antibiofilm EPS produced by yeast of the genera *Rhodotorula*. All the bacterial strains tested in this study were susceptible to the inhibitory activity of the EPS, particularly; the bacterial growth and biofilm formation of *Staphylococcus aureus* were inhibited in a higher degree. Since *S*. *aureus* is one of the most important pathogens in human health, the EPS could be formulated as an antiadhesive and antibiofilm treatment for medical and non-medical applications.
